# An X-ray Tomographic Study of Rechargeable Zn/MnO_2_ Batteries

**DOI:** 10.3390/ma11091486

**Published:** 2018-08-21

**Authors:** Markus Osenberg, Ingo Manke, André Hilger, Nikolay Kardjilov, John Banhart

**Affiliations:** 1 Institute of Material Science and Technologies, Technical University Berlin, Hardenbergstraße 36, 10623 Berlin, Germany; hilger@helmholtz-berlin.de (A.H.); banhart@helmholtz-berlin.de (J.B.); 2 Helmholtz-Centre Berlin for Materials and Energy GmbH, Hahn-Meitner-Platz 1, 14109 Berlin, Germany; manke@helmholtz-berlin.de (I.M.); kardjilov@helmholtz-berlin.de (N.K.)

**Keywords:** alkaline manganese batteries, X-ray tomography, in operando, in situ, zinc powder

## Abstract

We present non-destructive and non-invasive in operando X-ray tomographic investigations of the charge and discharge behavior of rechargeable alkaline-manganese (RAM) batteries (Zn-MnO_2_ batteries). Changes in the three-dimensional structure of the zinc anode and the MnO_2_ cathode material after several charge/discharge cycles were analyzed. Battery discharge leads to a decrease in the zinc particle sizes, revealing a layer-by-layer dissolving behavior. During charging, the particles grow again to almost their initial size and shape. After several cycles, the particles sizes slowly decrease until most of the particles become smaller than the spatial resolution of the tomography. Furthermore, the number of cracks in the MnO_2_ bulk continuously increases and the separator changes its shape. The results are compared to the behavior of a conventional primary cell that was also charged and discharged several times.

## 1. Introduction

The development of new energy storage materials and systems is currently one of the most important challenges in materials research. Batteries play a crucial role in the future replacement of conventional mobile or stationary energy sources based on fossil fuels. However, batteries with high storage capacities and low weights are still by far too expensive. Furthermore, the general shortage in various resources puts constraints on the development of many battery types. Therefore, the development of cost-efficient production methods and use of easily accessible raw materials are key issues in battery research.

Zinc is a widely available and inexpensive material, and it is a candidate for future use in rechargeable batteries for mobile and stationary applications [1,2,3,4,5,6,7]. The well-known alkaline-manganese battery is still one of the most common types in use [1]. Reasons include their low self-discharge and environmental friendliness compared to other battery types. Such batteries are cheap to produce, maintenance-free, and safe compared to lithium-based batteries. In addition, in the charged state they provide a voltage of 1.5 V, which is higher than many other (e.g., nickel-metal hydride) batteries. Their main disadvantage is that they are normally designed as primary cells, i.e., they are not rechargeable.

Because of the many fundamental advantages of alkaline-manganese batteries, much effort has been put into developing and optimizing primary cells and, even more important for future applications, developing rechargeable alkaline-manganese batteries (RAM) [8,9,10,11,12,13]. Up to now, RAM still suffer from an unreliable cyclic behavior. Some individual batteries can be recharged up to 500 times, while others last only a few cycles.

In the past, various methods have been applied to study alkaline primary cells. For the investigation of the zinc particles, electron microscopy and optical microscopy have been used [14,15,16]. Preparation of the samples is very difficult because the oxidation and corrosion of Zn, and the carbonation of ZnO alter the structure of the material. Horn et al. have developed a dedicated preparation technique [14]. However, all these measurement techniques do not allow for an in situ study of the material inside the entire volume of the battery. Only the sectioned material is accessible.

Imaging techniques based on X-rays have been successfully used to study battery materials [17,18,19,20,21,22,23,24,25,26,27,28,29]. Since these techniques are non-destructive and non-invasive, they are especially suited for in situ or in operando measurements [30,31,32,33]. X-ray tomography using both table-top and synchrotron radiation sources was used to investigate alkaline primary cells and zinc-air batteries in three dimensions [34,35,36,37]. Moreover, neutron imaging has been used to investigate alkaline primary cells [34,38].

In this paper, structural changes in RAM cells were examined in situ and non-destructively by X-ray tomography.

## 2. Experimental Set-Up and Data Processing

### 2.1. The Alkaline-Manganese Battery

#### 2.1.1. Set-Up

The alkaline-manganese battery consists of a steel shell into which the hollow cylinder of the cathode material—consisting of manganese dioxide and an electrolyte—was inserted by the manufacturer. The anode was made of a mixture of zinc powder and an electrolyte, and it was injected into the shell. Between the anode and the cathode, a separator is located. A metallic nail at the bottom of the battery acts as the negative pole of the battery. It protrudes into the anode and acts as a charge collector. Between the bottom and the cathode, a seal prevents leakage of the cell.

#### 2.1.2. Chemical Processes in an Alkaline-Manganese Battery

During the initial discharge, a reduction reaction takes place at the cathode; see Equations (1) and (2) [1]:

(1) $${\mathrm{MnO}}_{2}+H_{2}O+e^{-}\to \mathrm{MnOOH}+{\mathrm{OH}}^{-},$$

(2) $${3\mathrm{MnOOH}}_{2}+e^{-}\to {\mathrm{Mn}}_{3}O_{4}+{\mathrm{OH}}^{-}+H_{2}O$$

Due to the formation of MnOOH, the cathode expands in volume by about 17%. At the anode, as given in Equation (3), zinc initially forms zincate. After the electrolyte is supersaturated with zincate, the reaction product changes to zinc hydroxide, see Equation (4), which is then slowly dehydrated to zinc oxide, see Equation (5):

(3) $$\mathrm{Zn}+{4\mathrm{OH}}^{-}\to {\left[\mathrm{Zn}(\mathrm{OH}{)}_{4}\right]}^{2-}+2e^{-}$$

(4) $$\mathrm{Zn}+{2\mathrm{OH}}^{-}\;\to \mathrm{Zn}(\mathrm{OH}{)}_{2}+2e^{-}$$

(5) $$\mathrm{Zn}(\mathrm{OH}{)}_{2}\to \mathrm{ZnO}+H_{2}O$$

The overall discharge redox reaction is shown in Equation (6) [1]:

(6) $${2\mathrm{MnO}}_{2}+\mathrm{Zn}+2H_{2}O\to 2\mathrm{MnOOH}+\mathrm{Zn}(\mathrm{OH}{)}_{2}$$

For a small to medium discharge, the reaction in Equation (7) predominantly takes place in alkaline-manganese batteries [8]:

(7) $${3\mathrm{MnO}}_{2}+2\mathrm{Zn}\to 2{\mathrm{Mn}}_{3}O_{4}+2\mathrm{ZnO}+\mathrm{Zn}(\mathrm{OH}{)}_{2}$$

#### 2.1.3. Setup for Charge and Discharge of Alkaline-Manganese Batteries

The batteries were discharged with a VOLTCRAFT Multicharger VC 1506 that was connected to a computer. The batteries were charged using the charger type ACP62 PowerSet AA. For measuring the behavior of the charger, an oscilloscope was connected in parallel to a battery. To avoid damage, the conventional primary cells were charged in a pulsed mode and not to above 1.72 V. RAM were discharged to 0.9 V at currents of 100 mA, 200 mA, and 400 mA. The primary cell was discharged at 200 mA current.

#### 2.1.4. X-ray Tomography System and Measurement Procedure

The setup consisted of a fixed Hamamatsu microfocus X-ray tube (L8121-3) with a stable spot size of 7 µm and a Hamamatsu flat panel detector (C7942SK-05). The X-ray tube had an operational voltage range of 40 kV to 150 kV, and the target spot had a diameter of 7 µm [39]. The detector comprised a gadolinium oxysulfide (Gadox)-based scintillator on a 2316 × 2316 pixel detector array with a pixel size of 50 µm. On a goniometer, a sample can be mounted and moved with 5 degrees of freedom.

To avoid image analysis on large zinc-free spaces, cells with a homogenous zinc distribution inside the field of view were preferred. For that, multiple RAM and multiple primary cells were radiographed. Batteries were discarded if air inclusions were visible. Eventually, one primary cell and three RAM were selected. Before and after the first, second, third, fifth, 10th and if possible, 15th, charging step a tomogram was recorded.

For tomography, a source–object distance of 58 mm, and a source–detector distance of 350 mm were selected, which resulted in an effective pixel size of 8.3 µm, and thus a special resolution of 16.6 µm. The magnification chosen in this way was the largest that projected the entire image onto the detector, and not just a part of it. To achieve maximum contrast and the best signal-to-noise ratio, the X-ray tube was operated at 130 kV and 76 µA with a 0.5 mm copper filter. Furthermore, an exposure time of 1.6 s for each of the in total 1500 projections over 360° was applied, resulting in a total scanning time of 1.8 hr per tomogram. After image acquisition, the images were reconstructed using the software ‘Octopus’ (version 8, XRE, Gent, Belgium).

### 2.2. Data Processing

**Figure 1:**
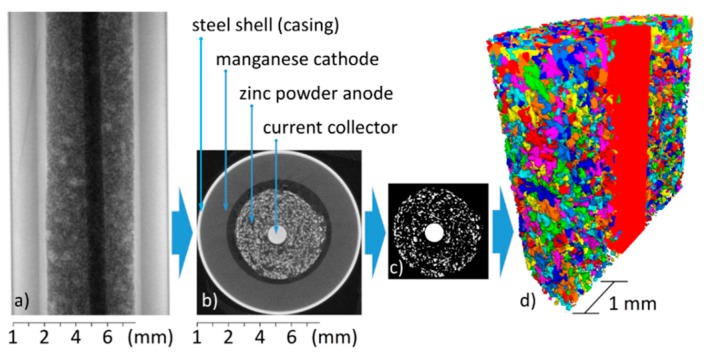
Sketch illustrating the reconstruction and data preparation process. After capturing all 1500 radiographic projection images—one shown in (**a**)—a tomographic 3D data set is reconstructed in (**b**). After binarization (**c**), the individual zinc particles are labelled (and, for example, color-coded as in (**d**)), which allows for a shape analysis of each individual particle.

For particle analysis, it is usually necessary to filter the data, because otherwise individual particles touching each other would be counted as a single particle, or noise artefacts would be interpreted as small particles. The choice of the filter thus had a strong impact on the significance of particle analysis. The reconstructed 3D data were filtered with the Software ‘Fiji’ (version 1.52a) [40,41], and then analyzed with the software ‘Avizo’ (version 8.1, Thermo Fisher Scientific, Waltham, MA, USA). Figure 1 demonstrates some of the main steps of the measurement and image analysis procedure schematically.

#### 2.2.1. Median Filter

Median filtering consists of first sorting all voxels to be analyzed, and their neighbors with respect to their grey values, and then lining them up in a list. The voxel to be analyzed then receives the grey level located in the middle of this list (i.e., the median value of all voxels in the neighborhood). With this method, noise is partially eliminated.

#### 2.2.2. Threshold Filter/Binarization

The 3D data sets were binarized with a threshold filter [42]. If a voxel of the dataset belonged to a zinc particle, the value 1 was assigned to it, whereas all other voxels received the value 0. After setting a threshold, all voxels above this value were set to 1 and all others to 0. The choice of the threshold value was crucial in this stage. The larger this threshold was chosen, the higher the X-ray absorption of a voxel had to be, to qualify as belonging to a particle.

#### 2.2.3. Erosion/Dilation

Eroding removes the edge/outer shell of a particle [42]. Mainly ‘noise dots’ (single voxels), but also very small particles that result from the recording process—for example, intersecting streak artefacts—and that do not belong to particles, disappear completely. Subsequent dilatation then adds the missing edges to the particles, but not to the now missing noise dots. In this way, the particles largely regain their previous volume, but the noise dots are removed. However, previously sharp particle edges tend to be smoothened.

#### 2.2.4. Watershed Transformation

To carry out a watershed transformation, a function is initially used to assign a distance from the particle edge to each voxel of a particle. If one now interprets the equidistance lines as water level lines after fictitious flooding with water, the volume is divided into several pools. If one fills them up with virtual water, one first obtains several smaller lakes that are merged to a larger lake at a watershed. Along this watershed, the particle is divided into two [42].

#### 2.2.5. Location Retrieval

In order to follow the dissolution process of individual particles, it was necessary to retrieve the location of the particles after each discharging or charging step. Therefore, a distinctive particle was chosen for each battery in the corresponding data set. After each cycle, this particle was located to ensure that the same areas were examined in the batteries. These particles were selected from areas neither directly next to the nail, nor to the separator, as it has been suggested that in these regions that a typical particle disintegration may occur.

In the image corresponding to the RAM discharged at 100 mA current for 10 cycles, it was no longer possible to find the initially selected particle. However, by comparing a number of other areas in this data set (around the initial particles) the original location of the particle was successfully reconstructed.

## 3. Experimental Results and Discussion

### 3.1. Properties of the Cells at Different Discharge Rates and Numbers of Cycles

**Figure 2:**
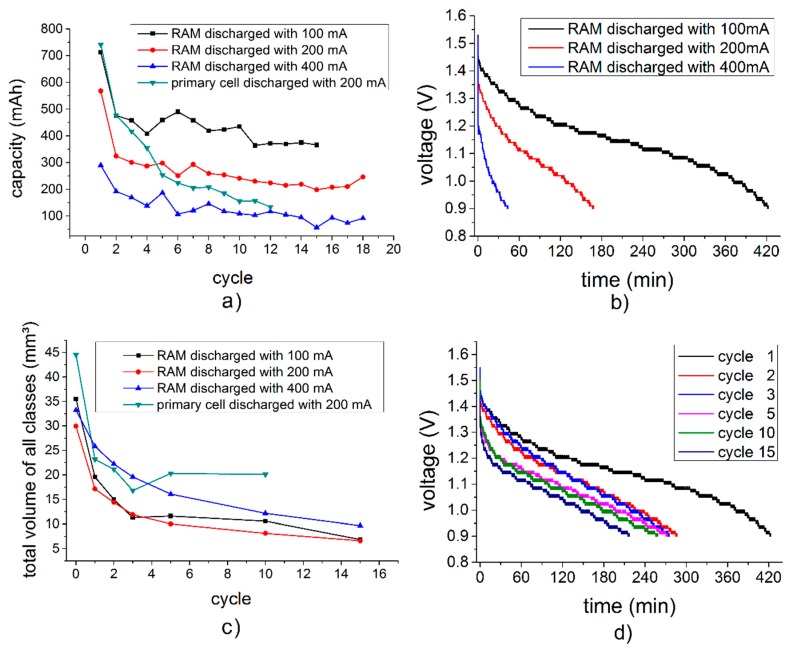
(**a**) Battery capacities remaining after a given number of discharge cycles, (**b**) discharge curves of pristine rechargeable alkaline-manganese (RAM) batteries at different discharge currents, (**c**) total volume of all segmented zinc particles and (**d**) discharge curves of a RAM battery (100 mA) during different cycles.

Figure 2a shows measured capacities of the individual cells per discharge/charge cycle. The final discharge voltage was 0.9 V. According to the manufacturer, the RAM should deliver up to 800 mAh, while the primary cell should deliver up to 1220 mAh at a discharge current of 30 mA.

It can be seen in Figure 2b that a higher discharge current results in a faster drop of voltage, which is associated with a lower discharge capacity after the cycle, and an increased internal resistance. The voltage also decreases with an increasing amount of cycles (Figure 2d). Of course discharging with lower currents also takes longer, as shown in the discharge curves in Figure 2b for the three different discharge currents applied in our work, namely 100 mA, 200 mA, and 400 mA.

Additionally a difference in the dissolving behavior of the zinc particles can be seen in Figure 2c. While the zinc particles in the RAM cells dissolve continuously during cycling, the zinc particles in the primary cell stopped dissolving after the third cycle.

### 3.2. 3D Structural Analysis

**Figure 3:**
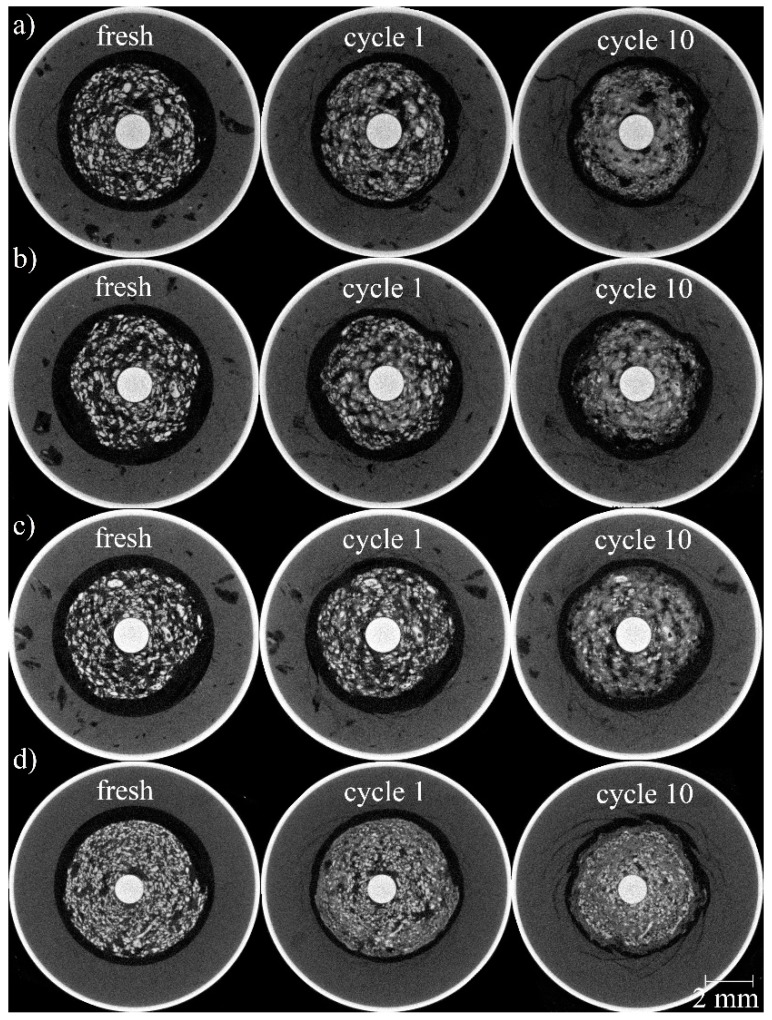
Tomographic cross sections showing three different cycle states for each of the four batteries studied. (**a**) RAM battery discharged at a current of 100 mA, (**b**) 200 mA, (**c**) 400 mA, and (**d**) alkaline manganese primary cell discharged at a current of 200 mA.

Figure 3 shows tomographic cross sections through samples in different charging stages. The cross sections of the fully charged and partially discharged batteries were always taken at the same locations (Section 2.2.5). After a first discharge, many of the smaller zinc particles were dissolved in the electrolyte (compare Figure 3, first and second column). Dissolution continuously progresses until most of the zinc particles have been dissolved into small particles that form a homogeneous gel in the electrolyte (i.e., particles are smaller than the spatial resolution of the tomography setup).

**Figure 4:**
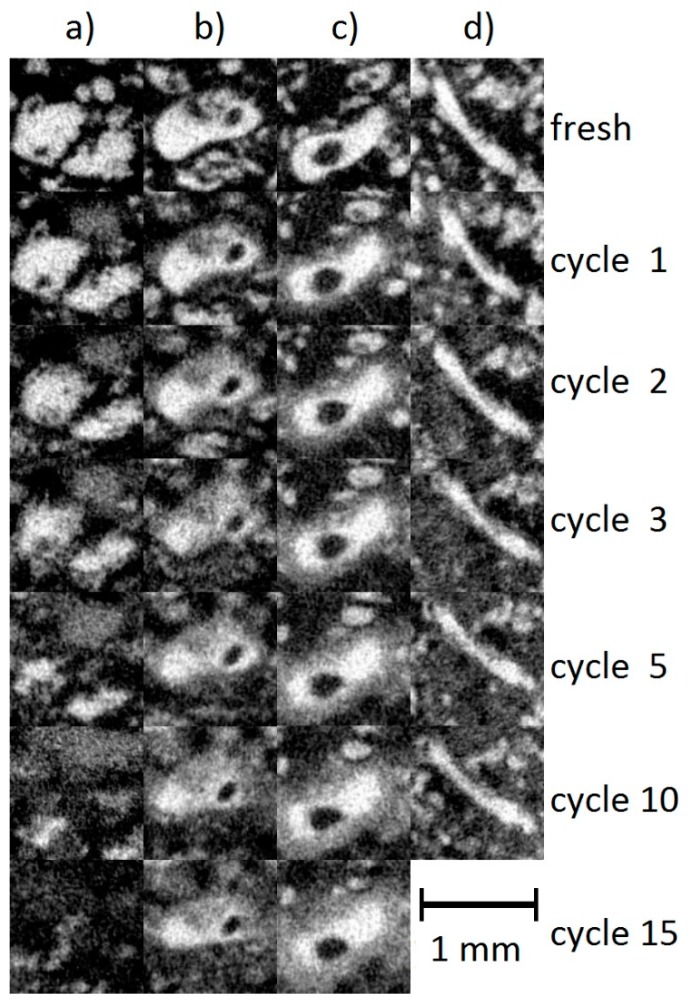
Overview over four selected particles in (**a**) RAM battery discharged with 100 mA, (**b**) 200 mA, (**c**) 400 mA, and (**d**) primary alkaline-manganese cell discharged with 200 mA.

However, in the images describing the RAM cells (Figure 3a–c), larger particles, especially at the outer ring area, still remained even after 10 cycles (see also Figure 4). Their size decreased, but their inner core remained almost unchanged. This reveals a layer-by-layer dissolving behavior (see also Figure 4). On the (outer) cathode side, the cylinder comprising the MnO_2_ and electrolyte slowly swelled and moves inwards, while at the same time cracks formed. Especially in the primary cell, the deformation resulted in the development of pointy structures in the separator region between the electrodes. The gap between both electrodes that was filled by the separator became smaller.

### 3.3. Dissolution of Individual Particles During Cycling

Cross sections through individual large particles occurring in each of the four tomographic measurement series showed the shapes of the particles after several cycling steps (Figure 4). Due to their size and good recognizability, even after several cycles, these particles were also used as “markers” for the location adjustments made when recording the data for Figure 3, i.e., they were used to find the same locations in the cell after different cycling steps. Obviously, the particles maintained their shapes over several cycling steps. However, the particle size slowly decreased and an area around the particles occurred that appeared fuzzy and in intermediate (grey) contrast, possibly containing small zinc particles that were no longer resolved by our measurement technique.

### 3.4. Quantitative 3D Analysis

**Figure 5:**
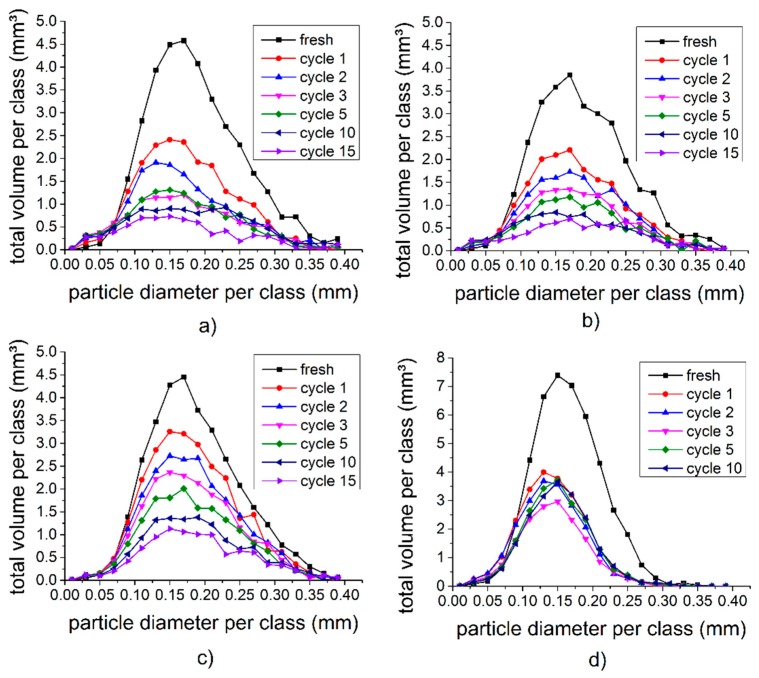
Particle size distributions in RAMs discharged at (**a**) 100 mA, (**b**) 200 mA, and (**c**) 400 mA and (**d**) primary cell discharged at 200 mA.

For a quantitative analysis of the zinc particles in the batteries, the air surrounding the battery, the steel casing, and the manganese dioxide cathode were “cut off”, as shown in Figure 1c, so that the respective image data contained only the anode material and the nail in the center. To this data set, the threshold filter was applied, after which the particles were white, and particle-free areas were left black. The same threshold was applied to all batteries and for all cycles. To reduce the noise in both the white and black areas, a median-3D filter was applied to the data sets (Section 2.2.3). Using the distinctive particles shown in Figure 4 as a starting point, the locations of 600 layers in each battery tomogram were adjusted to the same position, and the resulting data sets were quantitatively analyzed. This procedure ensures that exactly the same volume range for all cycling states of the battery were analyzed and any possible drift was eliminated After this, a watershed transformation was applied to improve particle separation (Section 2.2.4) using the program ‘Avizo 7.0’. For the radius-dependent analysis, each particle coordinate was converted from Cartesian to cylindrical coordinates, with the nail as a center. It was taken into account that the nails stuck askew in the batteries. The graphs in Figure 5 present the results of this particle size analysis. The particle diameter represents the corresponding spherical diameter. Figure 5a,c show the results for the RAM cell discharged at either 100 mA or 400 mA, while Figure 5b,d show the corresponding results for the RAM cell and the primary cell after discharging at 200 mA. Typical particle diameters range from 100 µm to 200 µm for both battery cell types. In all four cases, a rapid drop in the overall particles volume was found after the first discharge/charge cycle. This indicates that large zinc amounts were dissolved in the electrolyte, and that many particles reached a size where they could no longer be resolved by tomography and therefore did not contribute to the overall volume in the graphs shown. After each cycle, the volume found in the analyzed (larger) particles decreases in accordance to the findings in Figure 3. The size distributions of the particles remained almost unchanged.

One might expect that all particles shrink continuously. However, Figure 3 demonstrates that many particles dissolved quickly and “disappeared”, the smaller particles did so even faster than the larger ones. On the other hand, many larger particles became smaller and/or broke up, and they contributed to the amount of smaller particles while at the same time, several larger particles seemed to be largely unaffected by the cycling process. Eventually, the remaining particles seemed to have a size distribution that was very similar to the initial one, although many individual particles changed their size.

Figure 4 and Figure 5 reveal that particles dissolved more rapidly at lower than at higher discharge currents. Especially, the first discharge had a large effect. From the second discharge, the dissolution process was much slower and decreased continuously with increasing cycle numbers. During the discharge, the edges of large particles, dissolved and a shell with a lower absorption coefficient was formed around the particle. During charging, these clouds became smaller again. Very small particles were completely dissolved. This process was more noticeable near the separator.

Furthermore, during charging some very small new particles are formed, indicating the development of seeds for the growth of zinc crystals (see Supplementary Video 1).

### 3.5. Local Effects

The changes in particle sizes were not uniformly distributed over the entire cell. With progressing cycling, increased particle migration was observed, the distance and direction of which depended on the distance of the individual particle to the current collector nail. The particles in the RAM cell appeared to drift away from the collector nail towards the separator. This was observed only in the fifth cycle or later.

**Figure 6:**
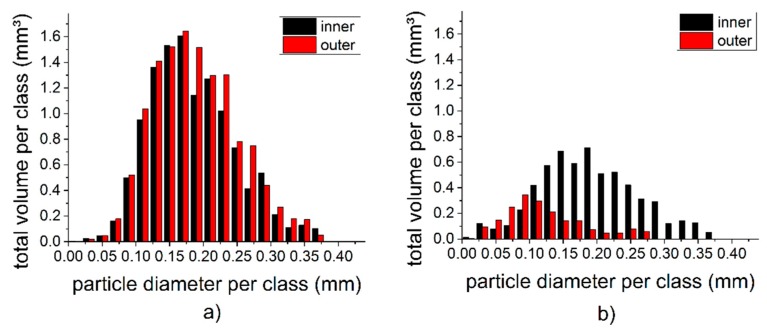
Size distributions of the zinc particles in the discharged RAM cell. (**a**) Pristine and (**b**) after the 10th cycle (discharge current 200 mA).

In the primary cell, in contrast, it seemed that starting from the first cycle, particles close to the nail migrated towards the center, while particles located close to the separator moved outwards, thus forming a ring with a lower particle density. The particle analyses of the pristine RAM cell and of the RAM cell after the 10th cycle, both discharged at 200 mA, as displayed in Figure 6. Here, the data set was divided into two annular disks (or in 3D hollow cylinders) of equal areas. The inner ring had a radius of 0.70 mm to 2.20 mm and the outer ring was 2.20 mm to 3.03 mm. The graphs show that with progressive cycling, the particles at a larger distance to the collector nail had less total volume and a smaller average particle diameter compared to other particles in the cell. Particle migration seemed to cause or at least contribute to a separation between the larger and smaller particles.

### 3.6. Comparison Between the Primary Cell and the RAM

As can be seen in Figure 5, a much larger volume of zinc particles was present in the primary cell (compare Figure 5d to Figure 5a–c). In addition, the maximum total volume per diameter class in the primary cell was observed for particles with 0.15 mm ± 0.02 mm diameter, which was slightly smaller compared to the 0.17 mm ± 0.02 mm for the RAM. Unlike the large particles in the RAM, the large particles of the primary cell dissolved faster than the smaller particles, thus leading to a shift of the average particle diameter upon cycling (Figure 5d).

**Figure 7:**
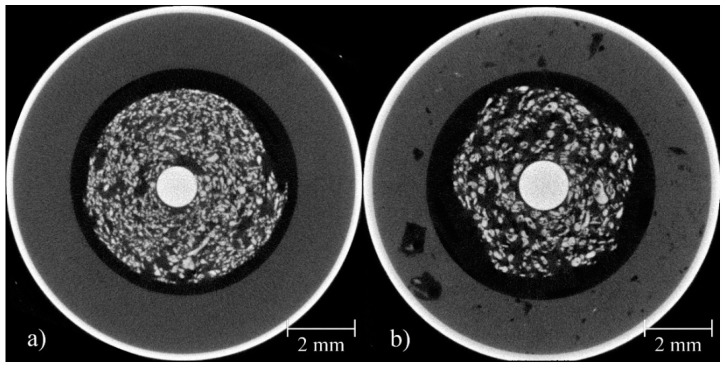
Comparative cut of the primary cell (**a**) and the RAM battery (**b**). Both cells are shown in their pristine state.

Direct comparison of slices taken from the RAM (Figure 7 bottom) and from the primary cell (Figure 7 top) revealed the different design of the two battery types. The graph in Figure 2c, representing the total volume of all particles, shows that the particle volumes declined sharply in both types of batteries after the first cycle. In the alkaline primary battery, the particle volume remained roughly constant in the ensuing cycles, whereas in the RAM it continued to decrease. This can be seen as well in Figure 5.

During the first discharge, the primary cell had the largest capacity (Figure 2a), which could be explained by the amount of zinc used. After their first cycle, all batteries lost a large part of their capacities. After 10 cycles, the capacities of the RAMs were still about 50% of their corresponding original capacity, while the primary cell reached only about 25% of the initial level. Furthermore, with an increasing number of cycles, the volume of the manganese dioxide cathode also increased, and the anode as well as the separator was increasingly compressed. This did not happen uniformly, but it created splinter-shaped structures that penetrated the separator. In the primary cell, this effect was much more evident. After the 12th cycle, the primary cell was no longer rechargeable and it began to leak.

**Figure 8:**
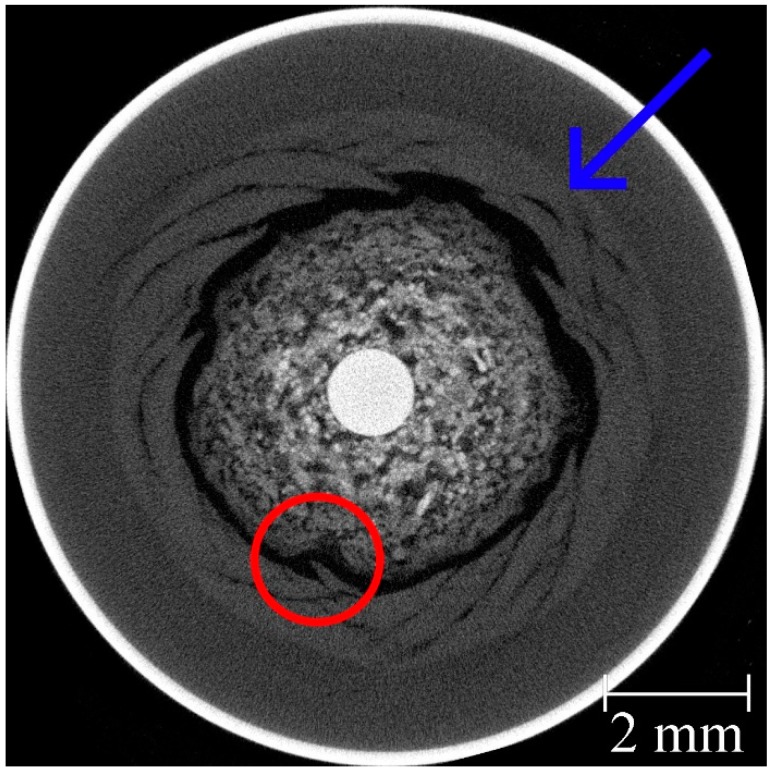
Tomographic cross section of the primary cell after 12 cycles.

After the failure of the primary alkaline battery, the tomogram exhibited some irregularities. Figure 8 demonstrates that many manganese dioxide spikes formed that pierced the separator (red circle). In addition, the manganese dioxide in the area around the separator was slightly brighter (blue arrow). This can be explained by a damaged separator so that zinc-enriched electrolyte could freely pass through to the cathode side. Therefore, the battery discharged itself and the manganese dioxide layer continued to expand, the internal pressure increased, and the battery started to leak. Presumably, in addition to the process of discharge, gas was formed, which may also have contributed to the increasing pressure [43].

## 4. Conclusions

By X-ray tomography, we have analyzed structural and morphological changes in rechargeable alkaline-manganese batteries, and in non-rechargeable primary cells, during repeated charge and discharge. We applied three different discharging currents (100 mA, 200 mA, and 400 mA) to the RAM cells. The size distributions of the zinc particles were calculated and compared (Figure 5). We found that first the smaller zinc particles disappear and after about 10 cycles also the larger ones dissolve. The degree and pace of dissolution differs between various locations in the cell. The zinc particles dissolve layer by layer and become increasingly smaller.

The structural changes in the primary alkaline cell are different than in the RAM cell. After the first discharge of the primary alkaline cell the overall zinc particle volume remains almost constant during cycling. Furthermore we found that new zinc particles are formed after cycling (see Supplementary Video 1). For the first few cycles, the primary alkaline battery performed well, and provided more capacity than the RAM battery. The total failure of the primary cell came suddenly and unexpectedly without any signs (see Figure 2a), may have been caused by separator penetration from one of the needle-like structures formed at the electrodes.

We think that the analysis and the corresponding results presented here can significantly contribute to the fundamental understanding and development of rechargeable alkaline batteries and of zinc-based batteries in general. In the future, similar studies might be done on other zinc-based systems such as zinc-air batteries, and they may contribute to the development not only of RAM cells with increased durability, but also of rechargeable zinc-air batteries.

## Supplementary Materials

The following are available online at http://www.mdpi.com/1996-1944/11/9/1486/s1, Video S1: Tomographic cross sections of the evolution of the RAM cell that was cycled with 200 mA.
